# N-glycomic profiling of capsid proteins from Adeno-Associated Virus serotypes

**DOI:** 10.1093/glycob/cwad074

**Published:** 2023-09-29

**Authors:** Yongjing Xie, Michael Butler

**Affiliations:** National Institute for Bioprocessing Research and Training, Foster Avenue, Mount Merrion, Blackrock, Co. Dublin, A94 X099, Ireland; National Institute for Bioprocessing Research and Training, Foster Avenue, Mount Merrion, Blackrock, Co. Dublin, A94 X099, Ireland; School of Chemical and Bioprocess Engineering, University College Dublin (UCD), Belfield, Dublin 4, D04 V1W8, Ireland

**Keywords:** adeno-associated virus, capsid protein, gene therapy, glycosylation, InstantPC

## Abstract

Adeno-associated virus (AAV) vector has become the leading platform for gene delivery. Each serotype exhibits a different tissue tropism, immunogenicity, and in vivo transduction performance. Therefore, selecting the most suitable AAV serotype is critical for efficient gene delivery to target cells or tissues. Genome divergence among different serotypes is due mainly to the hypervariable regions of the AAV capsid proteins. However, the heterogeneity of capsid glycosylation is largely unexplored. In the present study, the N-glycosylation profiles of capsid proteins of AAV serotypes 1 to 9 have been systemically characterized and compared using a previously developed high-throughput and high-sensitivity N-glycan profiling platform. The results showed that all 9 investigated AAV serotypes were glycosylated, with comparable profiles. The most conspicuous feature was the high abundance mannosylated N-glycans, including FM3, M5, M6, M7, M8, and M9, that dominated the chromatograms within a range of 74 to 83%. Another feature was the relatively lower abundance of fucosylated and sialylated N-glycan structures, in the range of 23%–40% and 10%–17%, respectively. However, the exact N-glycan composition differed. These differences may be utilized to identify potential structural relationships between the 9 AAV serotypes. The current research lays the foundation for gaining better understanding of the importance of N-glycans on the AAV capsid surface that may play a significant role in tissue tropism, interaction with cell surface receptors, cellular uptake, and intracellular processing.

## Introduction

**Fig. 1:**
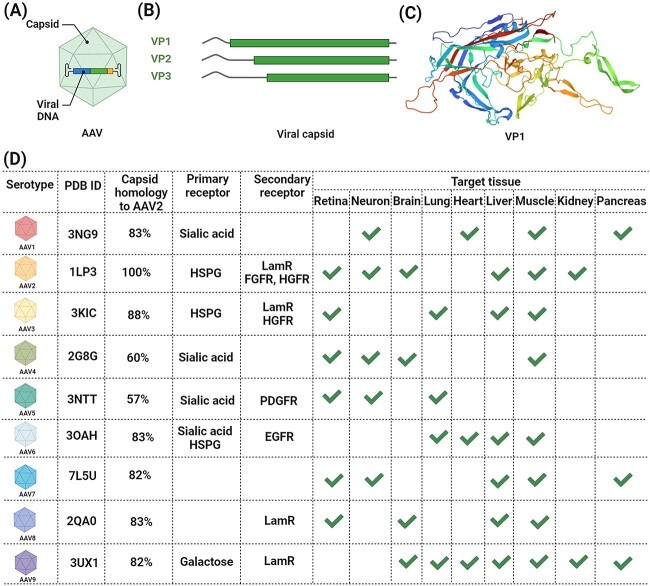
General information of the nine adeno-associated viruses (AAV) serotypes. A) The AAVs are viruses packaged with linear single-stranded DNA (ssDNA) and composed of non-enveloped capsids with T = 1 icosahedral symmetry. B) The AAVs are assembled from 60 viral proteins (VPs), including VP1, VP2, and VP3 in an approximate 1:1:10 ratio. The VPs share a common C-terminal that includes the entirety of VP3. C) The 3D structure of VP1 for AAV2 serotype. D) Summary and comparison of the PDB ID, capsid amino acid sequence homology to AAV2, primary receptors, secondary receptors, and target tissues for the nine AAV serotypes investigated in the current study. Created with BioRender.com.

First discovered in 1965 as a contaminant of adenovirus preparations (Atchison et al. 1965), the adeno-associated virus (AAV) vector has attracted significant attention and gradually become the leading platform for gene therapy (Goncalves 2005; Naso et al. 2017; Wang et al. 2019). As shown in Fig. 1, AAV is a small (25 nm in diameter), non-enveloped virus containing a linear single-stranded deoxyribonucleic acid (ssDNA) genome of approximately 4.8 kb (Daya and Berns 2008; Samulski and Muzyczka 2014). The genomic ssDNA is packaged into a near-spherical protein shell comprising 60 capsid protein subunits arranged with triangulation number (T) = 1 icosahedral symmetry, where T indicates the number of structural units per face of the icosahedron. Viral proteins (VPs) 1, 2, and 3 are the three key proteins and maintained in a molar ratio of 1:1:10 (Xie et al. 2002; Walters et al. 2004; Nam et al. 2007; Strauss and Strauss 2008). VP1 has a molecular weight (MW) of 81,945 Da and comprises 735 amino acids (UniProt accession NO: P03135-1). VP2 has a MW of 66,620 Da and comprises 598 amino acids (UniProt accession NO: P03135-2). Whereas VP3 has a MW of 60,063 Da and comprises 533 amino acids (UniProt accession NO: P03135-3; https://www.uniprot.org/uniprotkb). To date, 13 distinct primate AAV serotype vectors and numerous variants have been developed for gene delivery (Rayaprolu et al. 2013; Mietzsch et al. 2014; Pipe et al. 2019). Each exhibits a different in vivo tissue tropism or specificity, as well as a different genome capacity and safety profile (Zincarelli et al. 2008; White et al. 2011; Asokan et al. 2012; Lisowski et al. 2015; Srivastava 2016). Choosing a suitable AAV serotype with a specific tropism is critically important for preferential gene delivery to target cells or tissue types.

The precise mechanism of the tissue-tropism of the AAV serotypes remains unknown (Srivastava 2016), and the infectious pathway of AAV vectors is still not well understood (Summerford et al. 2016). Nevertheless, it has become increasingly clear that the host cell entry for most AAV vectors is mediated through initial attachment to specific cell surface glycans. This interaction typically involves sialic acid, heparan sulfate proteoglycan (HSPG), or galactose as primary receptors on the cell surface (Wu et al. 2006; Asokan et al. 2012; Lisowski et al. 2015; Srivastava 2016; Korneyenkov and Zamyatnin Jr 2021). Additionally, secondary receptors (or coreceptors) are also required for efficient AAV infection, that may include fibroblast growth factor receptor (FGFR), αVβ5 integrin, hepatocyte growth factor receptor (HGFR), platelet-derived growth factor receptor (PDGFR), laminin receptor (LamR), AAV receptor (AAVR) (Ding et al. 2005; Kashiwakura et al. 2005; Summerford et al. 2016; Korneyenkov and Zamyatnin Jr 2021). The characteristics of nine AAV serotypes investigated in the current study are summarized in Fig. 1. This includes information on Protein Data Bank entry identifier (PDB ID), capsid amino acid sequence homology to AAV2, primary receptors, secondary receptors, and target tissues obtained from the Research Collaboratory for Structural Bioinformatics Protein Data Bank (RCSB PDB, https://www.rcsb.org/) (Berman et al. 2000).

The structure of the AAV capsids can affect AAV vector-host cell interactions, including receptor binding, endocytosis, endosomal escape, intracellular trafficking, nuclear entry, uncoating, and antigenicity (Sanlioglu et al. 2001). Additionally, pseudotyping experiments demonstrated that the capsid proteins are exclusively responsible for tissue tropism (Rabinowitz et al. 2002; Grimm et al. 2006), which is dependent on the interaction between the capsid protein and cell surface receptors (Lisowski et al. 2015). The amino acid sequence of the AAV capsid proteins determines the structural dynamics and tissue tropism (Rayaprolu et al. 2013), and the capsid assembly is heterogeneous and divergent with variable composition (Worner et al. 2021). The AAV capsid homology among different AAV serotypes can vary substantially, in the range of 53%–99% (Daya and Berns 2008). The majority of sequence variation falls within the surface-exposed regions of the capsid, referred to as hypervariable regions (HVRs) (Wu et al. 2000; Govindasamy et al. 2006; Lochrie et al. 2006; Nam et al. 2007; Mays et al. 2013).

As one of the most prominent and diverse post-translational modifications (PTMs), protein glycosylation plays a critical role in cellular physiological and pathological function (Ohtsubo and Marth 2006; Reily et al. 2019). There are several types of glycans, including N-linked, O-linked, phosphorylated, glycosaminoglycans (GAGs), glycosphingolipids (GSLs), and glycosylphosphatidylinositol (GPI) anchors (Fuster and Esko 2005; Stanley 2011; Reily et al. 2019). The most abundant is the N-linked glycans with the attachment of β-N-acetylglucosamine (GlcNAc) to the side chain of asparagine (Asn or N) via an amide linkage, that comprises approximately 90% of the glycans present in eukaryotic glycoproteins (Apweiler et al. 1999; Varki 1999). The glycans on host cell surface contribute heavily to the dynamic interplay between virus and cells, such as viral entry, protection of proteolytic cleavage of viral proteins, recognition, and neutralization of viral capsid by host immune system (Raman et al. 2016; Miller et al. 2021). In addition to the glycans on host cell surface, PTMs of AAV capsid proteins are also known to regulate cell-type transduction preferences and viral infectivity (Nonnenmacher and Weber 2012; Mary et al. 2019). Of the 52 total PTMs detected in AAV1-rh10 serotypes, the abundance of glycosylation accounted for 36.53%, with phosphorylation, ubiquitination, SUMOylation, and acetylation in much lesser abundance at 21.15%, 17.30%, 13.46%, and 11.53%, respectively (Mary et al. 2019). Although there has been no previous research available to demonstrate the impact of AAV capsid glycoengineering, such as glycan removal, trimming, or modulation, on potential viral infection, mutations at the glycosylation sites or removal of N-linked glycans from similar virion proteins have been shown to interfere with viral entry, tissue specificity, and infectivity (Kuhn et al. 1988; Delos et al. 2002). Thus, detailed characterization and comparison of the glycosylation profiles of capsid proteins from different AAV serotypes is essential for better understanding the infection process and in the rational design of AAV vectors that exhibit selective or preferential tissue targeting. This would broaden the application of AAV as a platform for gene delivery. However, only the N-glycans of AAV 2 and 8 capsid proteins have been extensively identified in previous research. Although AAV serotypes 3, 5, 7, and 9 have also shown the presence of glycosylation, detailed information on their glycosylation profiles is unavailable (Murray et al. 2006; Aloor et al. 2018; Mary et al. 2019).

The aim of the current study was to address gaps in knowledge of AAV glycosylation by using our previously developed N-glycan preparation and profiling platform (Xie et al. 2021; Xie and Butler 2022) to characterize the N-glycomic profiles of nine AAV serotypes, 1–9. The streamlined workflow shortens the N-glycan preparation process substantially to approximately 10 min with good reproducibility (Xie et al. 2021). Additionally, the InstantPC label provides enhanced fluorescence sensitivity compared to other commonly used labels, such as 2-aminobenzamide (2-AB) and procainamide (Xie et al. 2021). This allows high-throughput and high-sensitivity N-glycomic profiling with detection of glycan species at low abundance. All the 9 AAV serotypes were expressed in suspension culture of human embryo kidney (HEK) Expi293F™ cells in serum-free, chemically defined media under identical conditions. Their N-glycosylation profiles were comparable, and a total of 35 major glycan peaks (GPs) containing 81 N-glycan structures were identified and compared. The most notable feature of the N-glycomic profiles was the high abundance of high-mannose N-glycans. These N-glycans (FM3, M5, M6, M7, M8, and M9) dominated the chromatograms, although the exact composition differed between AAV serotypes. Another feature was fucosylation and sialylation that existed in a much lesser abundance. This N-glycomic analysis lays a solid foundation to gain a better understanding of the significance of capsid N-glycosylation in AAV tissue tropisms, interaction with cell surface receptors, cellular uptake, and intracellular processing. The N-glycosylation profiles of the AAV capsid proteins may also be important for routine monitoring to ensure good consistency during AAV production and processing for gene delivery.

## Materials and methods

### Materials and reagents

The AdvanceBio Gly-X N-Glycan Prep with InstantPC kit, 96-ct (Part NO: GX96-IPC) consisting of three modules, including Gly-X deglycosylation module (Part NO: GX96-100), Gly-X InstantPC labeling module (Part NO: GX96-101), and Gly-X InstantPC Clean-up module (Part NO: GX96-102), AdvanceBio exoglycosidases of α (1-2,3,6)-Mannosidase (jack bean, Part NO: GKX-5010, JBM), Sialidase A (*Arthrobacter ureafaciens*, Part NO: GK80040, ABS), β (1-3,4)-Galactosidase (bovine testis, Part NO: GKX5013, BTG), β-N-acetylhexosaminidase (jack bean, Part NO: GK80050, GUH), and InfinityLab Ultrapure LC/MS acetonitrile (Part NO: 5191-4496) were donated by Agilent Technologies (Santa Clara, CA 95051, U.S.A.). α (1-2,4,6)-Fucosidase O (Omnitrophica bacterium, Part NO: P0749, FUCO) were purchased from New England Biolabs (Ipswich, MA 01938-2723, USA). LB Broth (Lennox) (Part NO: L3022-1KG) was purchased from Merck Life Science (Arklow, Co Wicklow, Ireland). Milli-Q water was used in all preparations. All the common chemicals were purchased from Merck Life Science (Arklow, Co Wicklow, Ireland).

### A‌AV vector plasmid constructs

The helper plasmid pAdDeltaF6 (Part NO: 112867, gift from James M. Wilson), Serotype specific Rep-Cap plasmids pAAV2/1 (Part NO: 112862, gift from James M. Wilson), pAAV2/2 (Part NO: 104963, gift from Melina Fan), pAAV2/5 (Part NO: 104964, gift from Melina Fan), pAAV2/7 (Part NO: 112863, gift from James M. Wilson), pAAV2/8 (Part NO: 112864, gift from James M. Wilson), and pAAV2/9n (Part NO: 112865, gift from James M. Wilson), and AAV-CMV-GFP (Part NO: 67634, gift from Connie Cepko) were purchased from Addgene (Watertown, MA 02472, U.S.A.). Rep-Cap plasmids of pAAV-RC3 Vector (Part NO: VPK-423), pAAV-RC4 Vector (Part NO: VPK-424), and pAAV-RC6 Vector (Part NO: VPK-426) were purchased from Cell Biolabs (San Diego, CA 92126, U.S.A.). The Plasmid was transformed into XL10-Gold Ultracompetent *Escherichia coli* cells (*E. Coli*, Part NO: 200315, Agilent Technologies, Santa Clara, CA 95051, U.S.A.) by heat shock method and the single colony was subsequently cultured in LB Broth (Lennox) medium containing 100 μg/mL ampicillin as described previously (Froger and Hall 2007; Green and Sambrook 2012). The purification of plasmid was carried out by using Invitrogen™ PureLink™ HiPure plasmid midiprep kit (Part NO: 10388702, Thermo Fisher Scientific, Waltham, MA, U. S. A.) according to the protocol supplied.

### Culture of human embryonic kidney Expi293F™ cells

Human embryonic kidney Expi293F™ cells (HEK Expi293F™, Part NO: A14527, Thermo Fisher Scientific, Waltham, MA, U. S. A.) were maintained in suspension in chemically defined, serum-free BalanCD HEK293 medium (Part NO: 91165-1 L, FUJIFILM Irvine Scientific, Co. Wicklow, Ireland) supplemented with 4 mM L-Glutamine (Part NO: 25030-081, Gibco-Fisher Scientific, Waltham, MA 02452, U.S.A.). The cells were cultured in Erlenmeyer flasks at 37 °C, 5% CO_2_ with agitation at 120 rpm in a humidified atmosphere. The viable cell density and cell viability was routinely measured by trypan blue exclusion staining (Strober 2015) on the LUNA-II Automated Cell Counter (Logos Biosystems, Villeneuve d’Ascq, France).

### Triple transfection for production of nine AAV serotypes

Triple transfection using suspension HEK Expi293F™ cells for the production of nine AAV serotypes were carried out by following the protocol described previously with minor modification (Grieger et al. 2016). One day before transfection, HEK Expi293F™ cells were seeded at 1.0 × 10^6^ cells/mL and incubated at 37 °C, 5% CO_2_ with agitation at 120 rpm in a humidified atmosphere. On the following day, cells were centrifuged at 1,500 rpm for 5 min and resuspended in suitable amount of medium to make final cell density at 2.2 × 10^6^ cells/mL, and aliquoted 27.0 mL into 125 mL Erlenmeyer flasks. Triple transfection was carried out with a total of 3.0 μg/mL of pAdDeltaF6, Rep-Cap plasmids of pAAV2/1, pAAV2/2, pAAV-RC3, pAAV-RC4, pAAV2/5, pAAV-RC6, pAAV2/7, pAAV2/8, pAAV2/9n, and AAV-CMV-GFP (molar ratio 1:1:1) and 1:2 weight ratio of transfection grade linear Polyethylenimine MAX ® (PEI MAX, MW 40000, Part NO: 24765, Polysciences, Warrington, PA 18976, U.S.A.) The three plasmids and the PEI MAX reagent were diluted with culture medium to make final volume as 1.5 mL each in two separate tubes, and then mixed and incubated for 20 min at room temperature. Subsequently, the mixture was drop-wise added to the cells, while gently swirling the Erlenmeyer flask. For production of AAV2 empty capsid, the transfection was carried out with a total of 3.0 μg/mL of pAdDeltaF6 and Rep-Cap plasmid pAAV2/2 without AAV-CMV-GFP, keeping the rest culture conditions identical. For production of AAV8 in the presence of added 40 mM KCl, extra 0.405 mL of 3.0 M KCl solution was added to the culture medium, keeping the rest culture conditions identical. Transfections were performed in biological triplicate.

### HEK Expi293F™ cell analysis and harvest

After 72 h post-transfection, viable cell density and cell viability was routinely measured. Additionally, transfection efficiency was determined by counting the green fluorescent protein (GFP) expression cells (EGFP filter cube: Ex470/30, Em530/50) against total cells stained with Hoechst 33,342 (DAPI filter cube: Ex375/28, Em460/50) on a CELENA ® X high content Imaging System (Logos Biosystems, Villeneuve d’Ascq, France). About 30.0 mL of each transfection was centrifuged at 1,500 rpm for 10 min, and the cell pellets and supernatants were stored at −80 °C for further analysis.

### HEK Expi293F™ cell process and AAV production

The cell pellets from 30.0 mL culture were resuspended in 6.0 mL of lysis buffer (50 mM Tris, 150 mM NaCl, 2 mM MgCl_2_, 0.5% polysorbate 20, pH 8.0), followed by three freeze/thaw cycles. After the last thaw, benzonase ® nuclease (Part NO: 70664-3, Merck Millipore, Arklow, Co Wicklow, Ireland) was added to the samples to a final concentration of 50.0 U/mL. The samples were incubated at 37 °C for 1.0 h with gentle agitation. After incubation, the samples were centrifuged at 13,000g for 10 min at 4 °C, and the supernatants were filtered through 0.45 μm filter and then stored at −80 °C until further analysis.

### Purification of AAV serotypes using AAVX affinity chromatography

Affinity chromatography purification of the nine AAV serotypes was performed by using POROS™ GoPure™ AAVX pre-packed column (Part NO: A36652, Thermo Scientific, Waltham, MA, U.S.A.) on the AKTA Avant System (GE healthcare, Chicago, Illinois, U.S.A.). The AAVX column was first equilibrated with binding buffer (25 mM sodium phosphate, pH 7.4) at a flow rate of 1.0 mL/min. After sample loading at lower flow rate of 0.5 mL/min and column washing with the binding buffer to remove unbound molecules, elution was performed using elution buffer (0.1 M glycine, pH 2.6) at the flow rate of 1.0 mL/min. The ultraviolet (UV) detector was set at both 280 and 260 nm. The collected fraction containing the target AAV serotypes was neutralized by adding 1/10 volume of 1.0 M Tris-HCl, pH 9.0, followed by buffer exchange as described below.

### Separation of AAV empty and full capsids using anion exchange chromatography

The AAV empty and full capsids were separated by anion exchange chromatography coupled to fluorescence and UV detection (AEX-FLD-UV) using Agilent 1290 Infinity II Bio UPLC system equipped with Bio strong anion exchange (SAX) column (NP5, particle size: 5 μm, particle type: non-porous, 4.6 mm × 250 mm, PEEK, Part NO: 5190-2467) under the control of OpenLab CDS software (Agilent Technologies, Santa Clara, California, USA). The UPLC system consists of binary solvent pump, autosampler, fluorescence detector, and UV–VIS detector. The fluorescence detector for intrinsic fluorescence of AAV capsid proteins was set with excitation and emission wavelengths at 280 nm and 350 nm, respectively. The UV detector was set at 260 and 280 nm. Buffer A was 70 mM 1,3-bis[tris(hydroxymethyl) amino] propane (BTP) containing 2 mM MgCl_2_ (pH 9.0), and buffer B was 70 mM BTP containing 2 mM MgCl_2_ and 250 mM sodium acetate (CH_3_COONa, pH 9.0). The AAV2 fraction after AAVX affinity chromatography purification and buffer exchange was injected at a volume of 10.0 μL. The column was equilibrated with 40% buffer B for 3 min, then a higher percentage of 60% buffer B was achieved from 3 to 5 min and held for another 10 min. Fraction containing AAV2 empty capsids without vector genomes was eluted and collected during this 12-min run. Even higher percentage of 100% buffer B was achieved from 15 to 17 min and held for another 10 min. Fraction containing AAV2 full capsids with CMV-GFP was eluted and collected during this 12-min run. Subsequently, buffer B was decreased to 40% from 27–28 min and equilibrated for another 7 min for the subsequent chromatographic separation cycle. The flow rate was set as 0.3 mL/min. Samples were maintained at 5 °C before injection, and the column temperature was 25 °C. For other AAV serotypes, the percentages of buffer B for elution of empty and full capsid differed and were adjusted accordingly.

### Determination the concentration and purity of AAV serotypes

The collected fractions of AAV serotypes were subjected to buffer exchanging and concentrating against 50 mM 4-(2-hydroxyethyl)-1-piperazineethanesulfonic acid (HEPES) buffer, pH 7.9, by using Pierce™ protein concentrator polyethersulfone (PES) with molecular weight cut off (MWCO) of 100 kDa (Part NO: 15566775, Fisher Scientific, Dublin 1, Ireland). The capsid protein concentration was determined using Pierce™ bicinchoninic acid (BCA) protein assay kit (Part NO: 23225, Thermo Scientific, Waltham, MA, U.S.A.) according to the kit protocol. The capsid protein concentration was also determined by UV spectrometry for the empty capsid (E = 1.7 for concentration in mg/mL) (Bennett et al. 2017). The purity of the AAVs was confirmed by sodium dodecyl-sulfate polyacrylamide gel electrophoresis (SDS-PAGE) with silver staining (Wray et al. 1981; Chevallet et al. 2006) using Invitrogen ™ SilverXpress ™ silver staining kit (Part NO: LC6100, Thermo Scientific, Waltham, MA, U.S.A.) and Coomassie Blue staining using Invitrogen ™ SimplyBlue ™ SafeStain (Part NO: LC6060, Thermo Scientific, Waltham, MA, U.S.A.) according to the kits protocol. The purified AAVs were then stored at −80 °C for subsequent analysis.

### InstantPC labeled N-glycans

The preparation of InstantPC labeled N-glycans from different AAV serotypes was carried out according to the manufacturer’s instruction (Agilent AdvanceBio Gly-X *N*-Glycan Prep with InstantPC kit, GX96-IPC). AAV samples (50.0 μg) were diluted with suitable amount of 50 mM HPPES buffer, pH 7.9, to make a final volume of 20.0 μL. Gly-X denaturant (2.0 μL) was added to the 20.0 μL of AAV solution, mixed thoroughly and incubated at 90 °C for 3 min. After leaving at room temperature for 2 min, 2.0 μL of *N*-glycanase (PNGase F: Peptide-N4-(acetyl-β-glucosaminyl)-asparagine amidase, EC 3.5.1.52, component from kit with Part NO: GX96-IPC, Agilent Technologies, Santa Clara, California, USA) working solution (*N*-glycanase: Gly-X digestion buffer 1:1 [v/v]) was added, mixed thoroughly, and incubated at 50 °C for 5 min. InstantPC dye solution was prepared by dissolving one vial of InstantPC dye (30 mg) with 150.0 μL of the accompanying solvent and mixed well. The InstantPC dye solution (5.0 μL) was added to the above prepared sample and incubated at 50 °C for 1 min. The load/wash solution (150.0 μL of 2.5% formic acid/97.5% acetonitrile) was added to each sample, and then the entire sample (179.0 μL) was transferred to each well of the Gly-X Clean-up plate containing 400.0 μL of the load/wash solution. After passing the solution through the clean-up plate by applying a vacuum, samples were washed with 600.0 μL of the load/wash solution for three times. InstantPC labeled N-glycans were eluted with 100.0 μL of Gly-X InstantPC eluent (160 mM ammonium formate/10% [v/v] acetonitrile, pH 4.4). The collected InstantPC labeled N-glycan solutions were dried by SpeedVac Vacuum Concentrator (Thermo Fisher Scientific, Waltham, MA, USA), and then analyzed immediately, or alternatively stored at −20 °C for future analysis.

### Exoglycosidase sequential digestion of InstantPC-labeled N-glycans

A total of ten tubes (each released from equivalent of 50.0 μg AAV6 capsid protein) of dried InstantPC labeled N-glycans prepared as described above were dissolved in 100.0 μL sequential digestion buffer (90.0 μL of water and 10.0 μL of 0.5 M ammonium acetate, pH 5.5). Exoglycosidases sequential digestion was accomplished by the addition of a panel of 2.0 μL α (1-2,3,6)-Mannosidase, α (2-3,6,8,9)-Sialidase A, β (1-3,4)-Galactosidase, β-N-acetylhexosaminidase, and α (1-2,4,6)-Fucosidase O to 10.0 μL of the above InstantPC-labeled N-glycans solution. Suitable amount of water was added to ensure the final volume as 20.0 μL in each reaction. The reactions were incubated at 37 °C for 24 h with gentle shaking. The enzymatic digested InstantPC-labeled N-glycan samples were dried by SpeedVac Vacuum Concentrator (Thermo Fisher Scientific, Waltham, MA, USA), and dissolved in 10.0 μL of Gly-X InstantPC eluent (160 mM ammonium formate/10% [v/v] acetonitrile, pH 4.4), then injected into the chromatographic system at a volume of 2.0 μL for analysis.

### InstantPC labeled N-glycan profiling by HILIC-FLD

The profiles of InstantPC labeled N-glycans or exoglycosidases sequential digestion mixtures were determined by hydrophilic interaction liquid chromatography coupled with fluorescent detector (HILIC-FLD) using Agilent 1,290 Infinity II Bio UHPLC system equipped with AdvanceBio Glycan Mapping column (300 Å, 1.8 μm, 2.1 mm × 150 mm, Part NO: 859700-913) under the control of OpenLab CDS software (Agilent Technologies, Santa Clara, California, USA). The fluorescence detector for InstantPC was set with excitation and emission wavelengths at 285 nm and 345 nm, respectively. The dried InstantPC labeled N-glycans were dissolved in 10.0 μL of Gly-X InstantPC eluent (160 mM ammonium formate/10% [v/v] acetonitrile, pH 4.4), and injected at a volume of 2.0 μL. The separation was carried out with 50 mM ammonium formate (pH 4.4) as solvent A and acetonitrile as solvent B. After initial system equilibrium for 5.0 min with 22% of solvent A and 78% solvent B (v/v) at a flow rate of 0.5 mL/min, the separation was carried out by a linear gradient of 78%–58% of solvent B (v/v) at a flow rate of 0.5 mL/min for 50 min, followed by a linear gradient of 58%–50% solvent B (v/v) at a flow rate of 0.5 mL/min for 10 min. Complete system equilibrium under 22% of solvent A and 78% solvent B (v/v) at a flow rate of 0.5 mL/min for another 15 min was necessary to ensure good chromatographic reproducibility. Samples were maintained at 5 °C before injection, and the separation column temperature was 60 °C. The system was routinely calibrated using AdvanceBio InstantPC Maltodextrin ladder (Part NO: GKPC-503, Agilent Technologies, Santa Clara, California, USA). The correlation between glucose unit (GU) value and chromatographic retention time was fitted to a fifth order polynomial function to obtain the standard curve.

### Verification of InstantPC labeled N-glycan structures by HILIC-FLD-ESI-MS/MS

The molecular mass, composition and sequence information of InstantPC-labeled N-glycans and exoglycosidases sequential digestion mixtures were verified by hydrophilic interaction liquid chromatography with fluorescence detection coupled with electrospray ionization mass spectrometry (HILIC-FLD-ESI-MS/MS) using Q Exactive Plus (Thermo Fisher Scientific, Waltham, Massachusetts, USA) equipped with Acquity UPLC Glycan BEH Amide Column (130 Å, 1.7 μm, 1.0 mm × 150 mm, Part NO: 186004850, Waters Corporation, Milford, Massachusetts, USA). Samples were maintained at 5 °C before injection, and the separation temperature was 60 °C. The InstantPC-labeled N-glycans were injected at a volume of 10.0 μL. The UPLC detector was set with excitation and emission wavelengths at 285 nm and 345 nm for InstantPC, respectively. The separation was carried out with 50 mM ammonium formate (pH 4.4) as solvent A and acetonitrile as solvent B. After initial system equilibrium for 5.0 min with 22% of solvent A and 78% solvent B (v/v) at a flow rate of 0.15 mL/min, the separation was carried out by a linear gradient of 78%–58% solvent B (v/v) at a flow rate of 0.15 mL/min for 50 min, followed by a linear gradient of 58%–50% solvent B (v/v) at a flow rate of 0.15 mL/min for 10 min. The system was equilibrated between samples by 78% solvent B (v/v) at a flow rate of 0.15 mL/min for another 15 min to ensure reproducibility. The running condition for MS was positive mode, spray voltage of 3.40 kV, capillary temperature of 320 °C, aux gas heater temperature of 300 °C, sheath and sweep gas flow rate of 30 L/h and 10 L/h, respectively, scan range of 450–2,500 m/z and resolution of 70,000. The HILIC-FLD-ESI-MS/MS spectra were processed and visualized by Xcalibur software (Thermo Fisher Scientific, Waltham, MA, USA). The molecular masses, compositions, fragmentations, and N-glycan structures were assigned with the aid of GlycoWorkbench software (Ceroni et al. 2008) with monoisotopic molecular mass increment of InstantPC at 261.1477 compared to the free reducing end form of glycans.

### Batch correction and data pre-processing

The chromatographic glycan peaks from the HILIC-FLD analysis were processed with the built-in software for automated peak picking and integration. Individual glycan peaks were analyzed on the basis of the correlation between measured retention time and glucose unit (GU) values generated from the 5th order polynomial standard calibration curve against AdvanceBio InstantPC Maltodextrin ladder under identical conditions. The chromatograms were all separated in the same manner into 35 major glycan peaks (GPs) and the glycan structures were assigned and verified by exoglycosidases sequential digestion and HILIC-FLD-ESI-MS/MS (Xie et al. 2021; Xie and Butler 2022). The complete assignment of InstantPC labeled N-glycans for AAV capsid proteins is shown in Supplementary Information ST1. The glycan structures were represented by following the Symbol Nomenclature for Glycans (SNFG) system (Varki et al. 2015). In addition to the 35 directly measured GPs, 4 derived glycan subclass traits were calculated as described previously with minor modifications (Saldova et al. 2014; Pavić et al. 2018). The calculation formula is shown in Supplementary Information ST4. These derived glycan subclass traits averaged specific glycosylation features (sialylation, galactosylation, fucosylation, and mannosylation) across different individual N-glycan structures.

To remove experimental variation from measurements, batch correction and normalization were performed on glycan data. Total area under the curve normalization was applied, where the percentage (or relative abundance, %) of each glycan peak and subclass trait were calculated by the integrated peak area under the curve (AUC) of each glycan peak divided by AUC from total glycan peaks of the corresponding chromatogram (Abella et al. 2005). This represented the composition of glycans and subclasses in a AAV serotype sample. The equation was as follows:


\begin{align*} FLR\ AU C\left(\%\right)=&\left( FLR\ AU{C}_{Glycan\ i}/\mathrm{sum}\left( FLR\ AU{C}_{Glycan s}\right)\right)\\&\quad \mathrm{x}\ 100\ \% \end{align*}

### Statistical analysis

A significant difference among different AAV serotypes was tested with Two-way analysis of variance (ANOVA) (Zhang 2014) by using biological triplicates of all the AAV serotypes with GraphPad Software (San Diego, CA, USA.). To distinguish the similarity between different AAV serotypes to identify potential relationships, factor analysis, logistic regression model, principal component analysis (PCA), and hierarchical clustering heatmap under R environment with packages of blorr, FactoMineR, factoextra, pheatmap, ggplot2 (version 4.1.1) (Rizzo 2008) were used. This is a free software environment for statistical computing and graphics.

## Results

### Production, purification, and characterization of nine AAV serotypes

Nine AAV serotypes were produced by using helper-free transient triple plasmid transfection of human embryo kidney (HEK) Expi293F™ cells in suspension culture as described previously with minor revision (Ayuso et al. 2010; Chahal et al. 2014; Grieger et al. 2016; Su et al. 2020). As presented in greater detail in the Materials and Methods section, the whole workflow involved transformation of plasmid DNA into competent *E. coli* cells, plasmid expansion and purification, HEK Expi293F™ cell expansion, triple plasmid transfection, AAV production, purification, and characterization. Biological triplicate samples of each AAV serotype were produced under identical conditions. In order to ensure that variations in N-glycosylation profiles reflected inherent differences of the AAV serotypes rather than variations in AAV preparation, several critical steps were standardized. These included: (1) Each plasmid was produced from identical *E. coli* single colonies and purified in one batch (mg range). Because both helper (pAdDeltaF6) and AAV-CMV-GFP plasmids were required for production of nine AAV serotypes, a substantial larger quantity of these two plasmids were needed compared to individual Rep-Cap plasmids. Plasmid purity and integrity were analyzed by the ratio of UV absorbance 260/280 nm and 260/230 nm (Glasel 1995; Lucena-Aguilar et al. 2016). (2) In order to ensure consistent efficiency, transfection of HEK Expi293F™ cells was carried at identical cell passage numbers in a final volume of 30 mL. As shown previously, cell passage number affects the transfection efficiency (de Los Milagros Bassani Molinas et al. 2014). (3) PEI-DNA complex formation was controlled at 20 min at room temperature. The incubation time for the formation of these complexes has been shown to be critical with longer periods reducing efficiency for both HEK 293 cells (Gonzalez-Dominguez et al. 2022) and Chinese Hamster ovary (CHO) cells (Sang et al. 2015). (4) Affinity chromatography (Rodriguez et al. 2020) was used to purify all the AAV serotypes with AAVX affinity resin, followed by anion exchange chromatography (AEX) (Aebischer et al. 2022) for the separation of AAV empty and full capsids. This method allowed high specificity for each AAV serotype and high efficiency in clearance of impurities, high and low molecular weight species, host cell proteins (HCPs) and DNA. This overall method of AAV preparation compared favorably to the commonly used density gradient centrifugation (Vinograd and Hearst 1962; Vinograd et al. 1963).

### N-glycosylation characterization of the AAV serotypes

**Fig. 2:**
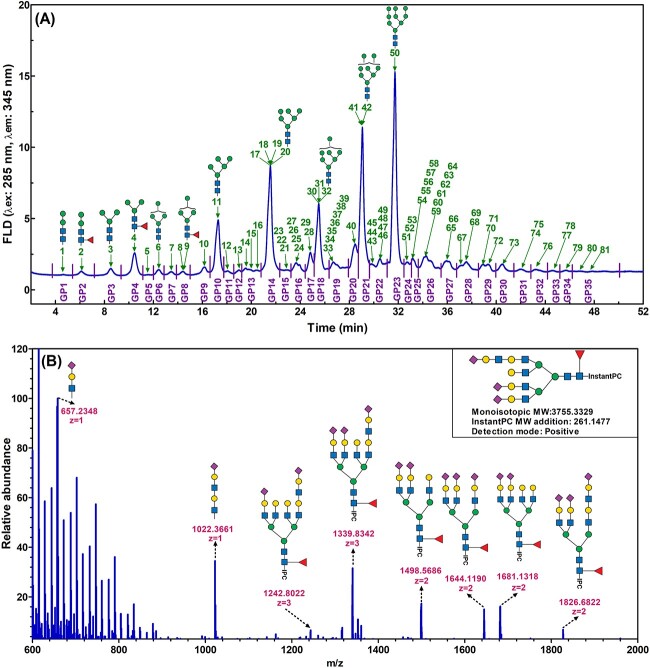
A) Representative HILIC-FLD chromatogram of InstantPC labeled N-glycans from AAV6 serotype. This shows fluorescence detection over a 52-min chromatogram. The peaks are identified as GP1 to GP35 and the N-glycan structures are numbered as 1 to 81. In some cases, several N-glycan structures were identified in a single glycan peak (GP), for example GP14 contains N-glycan structures of 17–20. Selective major abundant N-glycan structures are displayed. B) Representative ESI-MS/MS spectrum obtained from one of the N-glycan peaks in the HILIC-FLD chromatogram (GP31). The N-glycan structures contained in the HILIC-FLD chromatogram were assigned following ESI-MS/MS analysis, and it was identified as Hex8HexNAc7NeuAc4dHex1-InstantPC which was assigned as FA4G4Lac1S3. Trace N-glycan fragment derived from other N-glycan structures within GP31 are labeled. Symbol nomenclature: square: N-acetylglucosamine; green circle: mannose; yellow circle: galactose; triangle: fucose; diamond: N-acetylneuraminic acid. The complete dataset used for N-glycan structural assignment for the AAV capsid proteins is included in Supplementary Information ST1.

The N-glycosylation profiles of nine AAV serotypes were analyzed by hydrophilic interaction liquid chromatography with fluorescence detection (HILIC-FLD). Figure 2A shows a representative HILIC-FLD chromatogram of the InstantPC labeled N-glycans from AAV6 serotype. The 52-min chromatogram shows a separation of 35 major glycan peaks (GPs) that are identified as GP1 to GP35 according to retention time. These numbers are shown above the peaks (in green) in Fig. 2A as well as the assigned N-glycan structures. Each peak was subjected to electrospray ionization mass spectrometry (ESI-MS/MS) fragmentation to obtain structural information of the N-glycans.

The small peak of GP31 was selected as an example to demonstrate the detailed characterization of the N-glycan structure (Fig. 2B). It was confirmed to contain Hex8HexNAc7NeuAc4dHex1-InstantPC with a possible N-glycan structure assigned as FA4G4Lac1S3. The ability to assign an N-glycan structure to even a relatively minor peak demonstrated the high sensitivity of the current N-glycan preparation and profiling platform. The N-glycan structures were assigned by a combination of the ESI-MS/MS analysis and the previously established InstantPC N-glycan database which contains 264 distinct reference structures (Xie and Butler 2022). A full dataset of 81 N-glycan structures in all the nine AAV serotypes analyzed is presented in the Supplementary Information (ST1—AAV N-glycan assignments). Glycan linkages and isomers are not considered in the current study. Some N-glycan structures were identified but not included in the table due to their low abundance.

**Fig. 3:**
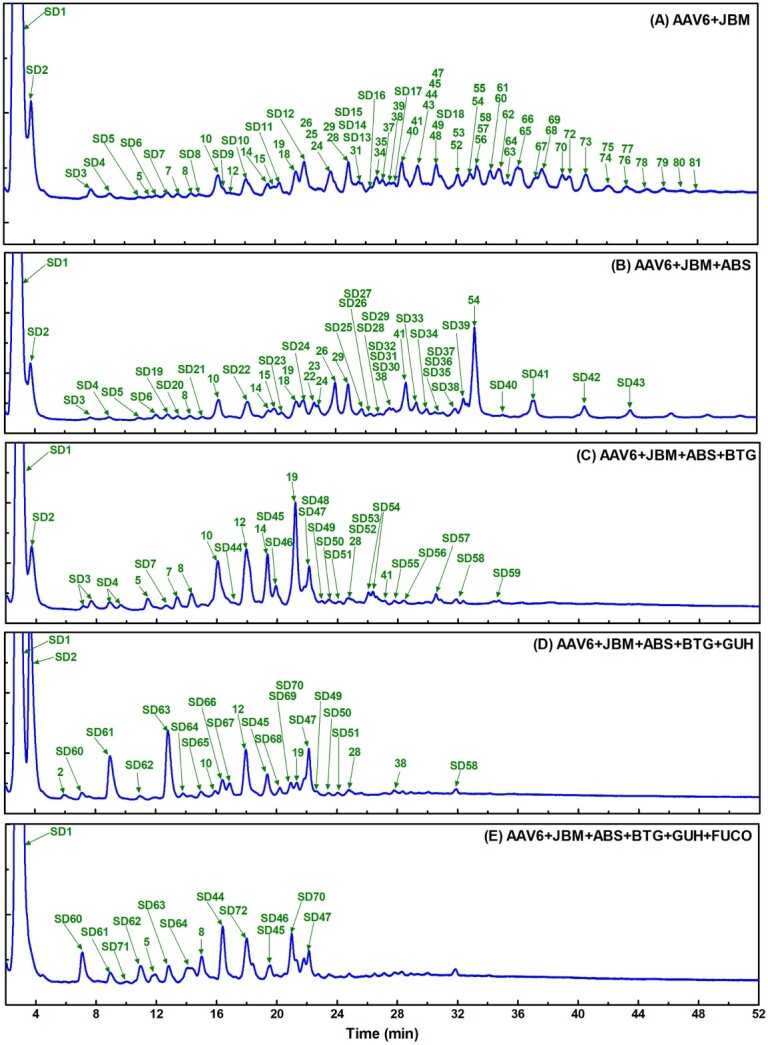
Representative exoglycosidases sequential digestion array for InstantPC labeled N-glycans from AAV6 serotype by HILIC-FLD. The initial N-glycan profile prior to digestion is displayed in Fig. 2A. N-glycan profiles subjected to specific exoglycosidase enzyme arrays are shown as follows: A) Αlpha (1-2,3,6)-mannosidase (Jack bean, JBM) to cleave terminal, non-reducing α (1-2,3,6)-linked mannose. B) Sialidase A (*Arthrobacter ureafaciens*, ABS) to cleave linear and branched terminal, non-reducing α2-3,6,8,9-linked sialic acid. C) β (1-3,4)-galactosidase (bovine testis, BTG) to cleave terminal β (1-3,4)-linked galactose. D) β-(1-2,3,4,6)-hexosaminidase (jack bean, GUH) to cleave terminal, non-reducing β-(1-2,3,4,6)-linked N-acetylglucosamine (GlcNAc) and N-acetylgalactosamine (GalNAc). Higher enzyme concentrations may be necessary to remove bisecting β-(1-4)-linked GlcNAc. E) α (1-2,4,6)-Fucosidase O (Omnitrophica bacterium, FUCO) to cleave α (1-2,4,6)-linked fucose, but it cleaves α (1-6) core fucose more efficiently than other linkages. The numbers shown above the peaks refer to the structures in the initial dataset in the Supplementary Information (ST1—AAV N-glycan assignments). The new N-glycan peaks after exoglycosidase sequential digestion are labeled as sequential digestion followed by numbers (SD1-72) and included in the secondary dataset in Supplementary Information (ST1—Exoglycosidase sequential digestion). The dataset contains 81 N-glycan structures identified in the analysis prior to digestion (1-81) and 72 new N-glycan structures identified as exoglycosidases sequential digestion (SD1-72).

To further confirm the structural assignments, InstantPC labeled N-glycan samples were subjected to exoglycosidases sequential digestion by an array of specific exoglycosidases to cleave specific monosaccharide residues with specific glycosidic linkages. The exoglycosidases sequential digestion was further verified by HILIC-FLD-ESI-MS/MS. The representative HILIC-FLD chromatograms for InstantPC labeled N-glycans from AAV6 serotype with a panel of exoglycosidase compositions to sequentially cleave mannose, sialic acid, galactose, N-acetylglucosamine (GlcNAc), and fucose are displayed in Fig. 3A–E, respectively. The exoglycosidases sequential digestion analysis enabled data to be obtained for glycan structures that were co-eluted. This analysis generated another 72 new N-glycan structures that were labeled as sequential digestion (SD) 1–72 in Fig. 3 with the complete dataset listed in Supplementary Information (ST1—Exoglycosidase sequential digestion). This method is based upon the known substrate specificity of a number of selected enzymes that have been used previously for analysis and presentation of complex pools of N-glycans from human serum (Saldova et al. 2014) and SARS-COV2 spike proteins (Xie and Butler 2023).

InstantPC labels N-glycans in a one-to-one molar ratio regardless of glycan structures and therefore the relative abundance (%) of the glycans can be correlated with the areas under the curve (AUCs) detected by fluorescence. We analyzed the N-glycosylation profiles of all nine AAV serotypes. They exhibited comparable N-glycosylation profiles with each glycan peaks eluted at a similar retention time but with variations in peak heights or areas. Of the 81 major N-glycan structures identified by HILIC-FLD, there were no specific N-glycan peaks exclusively present in or absent from any specific AAV serotype. As shown in Fig. 2A, GPs 4, 10, 14, 18, 21, and 23 were the most abundant peaks that represented 73% of the total N-glycans of AAV6 serotype. These six N-glycans were high mannose structures, namely FM3, M5, M6, M7, M8, and M9. The rest of the N-glycans were present at significantly lower abundance.

### Quantitative comparison of N-glycosylation profiles among different AAV serotypes

**Fig. 4:**
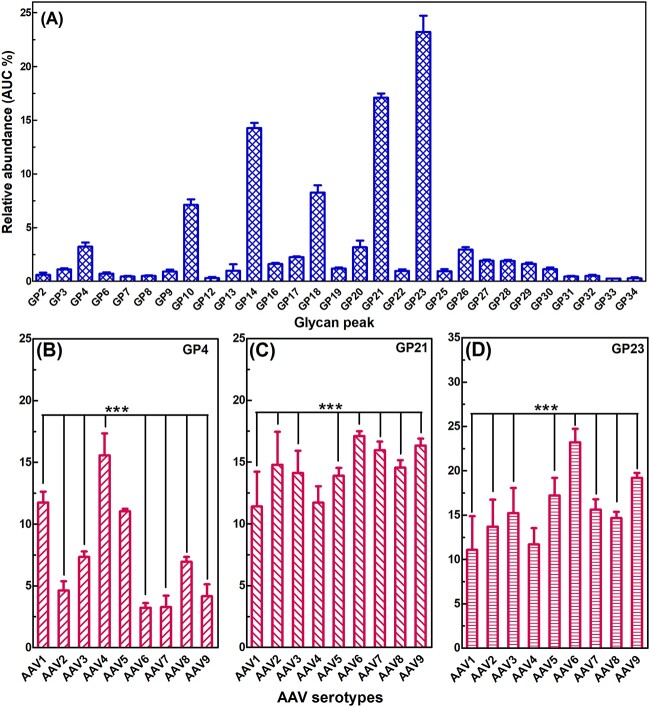
Variation in relative abundance (%) of N-glycans between AAV serotypes. A) Representative average relative abundance and sample standard deviation (AUC % ± SD) of major GPs generated from biological triplicates of AAV6 serotype. B) Direct comparison of relative abundance (%) of GP4 (FM3) between AAV serotypes. C) Direct comparison of relative abundance (%) of GP21 (M8) between AAV serotypes D) direct comparison of relative abundance (%) of GP23 (M9) between AAV serotypes. For ease of visualization, only the comparison of biological triplicates between AAV1 and other 8 AAV serotypes by two-way ANOVA is displayed, with * as *P* < 0.05, ** as *P* < 0.01, *** as *P* < 0.001. The complete dataset for relative abundance (%) of the nine AAV serotypes and statistical analysis by two-way ANOVA are shown in Supplementary Information ST2 and ST3.

Although the N-glycan profiles of the nine investigated AAV serotypes were comparable, we observed differences in the relative abundance (%) of individual glycan peaks (GPs). The relative abundance (%) of the major GPs for AAV6 serotype is displayed in Fig. 4A as a representative spectrum. Table 1 summarizes the assignment and relative abundance (%) of the abundant N-glycan structures. A complete dataset for biological triplicate samples of all the nine AAV serotypes is displayed in Supplementary Information (ST2—AAV GPs relative abundance). GPs 1, 5, 11, 15, 24, and 35 were excluded due to their low abundance. GPs 14 (assigned M6), 21 (M8), and 23 (M9) were the three most abundant peaks, accounting for 14.3 ± 0.5%, 17.1 ± 0.4%, and 23.2 ± 1.5% of the total N-glycans, respectively. GPs 10 (M5) and 18 (M7) exist at relatively lower abundance (<10%) at 7.1 ± 0.5% and 8.3 ± 0.7%. These 5 most abundant GPs accounted for approximately 70% of the total N-glycans. Additionally, GPs 4 (FM3), 20 (FA2G2S1), and 26 (M10 and FA3G3S2) accounted for approximately 3.0% each. This left the remaining 27 GPs that accounted for only about 20% of the total N-glycans.

**Table 1:** Assignment and relative quantification of the major abundant N-glycan structures identified in the AAV6 serotype.

Glycan peak	Relative abundance (%)		Monosaccharide composition	Determined N-glycan assignment
	Mean	SD		
GP2	0.605	0.216	Hex2HexNAc2dHex1	FM2
GP3	1.101	0.116	Hex3HexNAc2	M3
GP4	3.235	0.375	Hex3HexNAc2dHex1	FM3
GP6	0.721	0.120	Hex4HexNAc2	M4
GP7	0.455	0.064	Hex3HexNAc3dHex1	FA1 or FM3B
GP8	0.502	0.062	Hex3HexNAc4	A2
			Hex4HexNAc2dHex1	FM4
GP9	0.907	0.190	Hex3HexNAc4dHex1	FA2
GP10	7.114	0.509	Hex5HexNAc2	M5
GP13	0.980	0.608	Hex3HexNAc5dHex1	FA3 or FA2B
			Hex4HexNAc5	A3G1 or A2BG1
GP14	14.281	0.463	Hex6HexNAc2	M6
GP16	1.609	0.127	Hex5HexNAc5	A3G2 or A2BG2
			Hex5HexNAc4dHex1	FA2G2
GP17	2.265	0.060	Hex5HexNAc5dHex1	FA3G2 or FA2BG2
GP18	8.258	0.682	Hex7HexNAc2	M7
GP19	1.195	0.090	Hex5HexNAc4NeuAc1	A2G2S1
GP20	3.175	0.633	Hex5HexNAc4NeuAc1dHex1	FA2G2S1
GP21	17.109	0.378	Hex8HexNAc2	M8
GP22	0.977	0.143	Hex5HexNAc5NeuAc1dHex1	FA3G2S1 or FA2BG2S1
			Hex5HexNAc4NeuAc2dHex1	FA2G2S2
GP23	23.210	1.520	Hex9HexNAc2	M9
GP25	0.949	0.189	Hex7HexNAc6dHex1	FA4G4
GP26	2.957	0.222	Hex10HexNAc2	M10
GP27	1.917	0.111	Hex7HexNAc6NeuAc1dHex1	FA4G4S1
			Hex7HexNAc6NeuAc2dHex1	FA4G4S2
GP28	1.899	0.114	Hex7HexNAc6NeuAc2dHex1	FA4G4S2
			Hex7HexNAc6NeuAc3dHex1	FA4G4S3
GP29	1.625	0.122	Hex7HexNAc6NeuAc3dHex1	FA4G4S3
			Hex7HexNAc6NeuAc4dHex1	FA4G4S4
GP30	1.141	0.144	Hex7HexNAc6NeuAc4dHex1	FA4G4S4
GP31	0.454	0.058	Hex8HexNAc7NeuAc3dHex1	FA4G4Lac1S3
			Hex8HexNAc7NeuAc4dHex1	FA4G4Lac1S4
GP32	0.508	0.088	Hex8HexNAc7NeuAc4dHex1	FA4G4Lac1S4

(1) Monosaccharide codes: Hex: Hexose, HexNAc: Hexose with an N-acetylated amino group; dHex: Hexose without a hydroxyl group; NeuAc: N-Acetylneuraminic acid. (2) These are the major N-glycan structures found in the glycan peaks shown in Figs 2A and 4A. The full dataset from all 9 AAVs is displayed in the Supplementary Information ST1 and ST2.

Two-way analysis of variance (ANOVA) (Zhang 2014) was employed to evaluate statistical significance in the variation of the N-glycosylation profiles among different AAV serotypes. Comparisons were made between all combinations of the GPs from the analyzed AAV serotypes. The complete ANOVA data is displayed in Supplementary Information (ST3—ANOVA statistical analysis).

The three most variable peaks that demonstrated high statistical significance difference among the AAV serotypes are GPs 4 (FM3), 21 (M8), and 23 (M9). Therefore, these peaks were selected to show the variation in N-glycosylation profiles among the AAV serotypes in greater detail (Fig. 4B–D). As shown in Fig. 4B, serotype AAV4 possessed the highest GP4 (FM3) content at 15.6%, followed by AAVs 1 and 5 at 11.0%. The relative abundance of GP4 (FM3) in AAVs 3 and 8 was relatively modest at 7.0%, while this decreased to under 5.0% for AAVs 2, 6, 7, and 9. There was a significant difference in the relative abundance of GP4 (FM3) between AAV1 and its counterparts (*P* < 0.001), except AAV5 where no significant difference was observed (*P* > 0.05). Figure 4C showed that the relative abundance of GP21 (M8) in AAVs 1 and 4 was just under 12%, and there was no significant difference between these two serotypes (*P* > 0.05). The rest of the AAV serotypes, however, showed a significantly higher content of GP21 (M8) than AAV1 (*P* < 0.001). Figure 4D showed that the relative abundance of GP23 (M9) in AAVs 1 and 4 was just under 12.0%, and there was no significant difference between these two serotypes (*P* > 0.05). The rest of the AAV serotypes showed a significantly higher content of GP23 (M9) than AAV1 (*P* < 0.001). The relative abundance of GP23 (M9) for AAV6 was as high as 23.2 ± 1.5%. Among the other peaks, GPs 2 (FM2), 3 (M3), 10 (M5), and 14 (M6) demonstrated statistical significance between AAV serotypes but with a lower variability.

### Variations in AAV N-glycomic profile with changing HEK transfection conditions

A variety of improvements have been explored to increase both yield and quality of AAV production to meet the increasing demand of gene therapy. For example, previous studies have shown that adding specific concentrations of sodium chloride (NaCl), or potassium chloride (KCl), or a combination of both, to the production medium led to a substantial increase in AAV vector particles and infectious titres of herpes simplex virus (HSV) (Adamson-Small et al. 2017; Yu et al. 2021). Although the desired product of triple transfection of the HEK 293 cells is AAV containing full length of vector genome, this is often accompanied with the formation of “empty” capsids lacking the genomic material, and therefore are unable to provide a therapeutic benefit (Wright 2014a, 2014b).

One critical question is whether the encapsidation of genomic material into the AAV capsid shell or the presence of added salts to the culture medium affects its overall N-glycosylation profile.

**Fig. 5:**
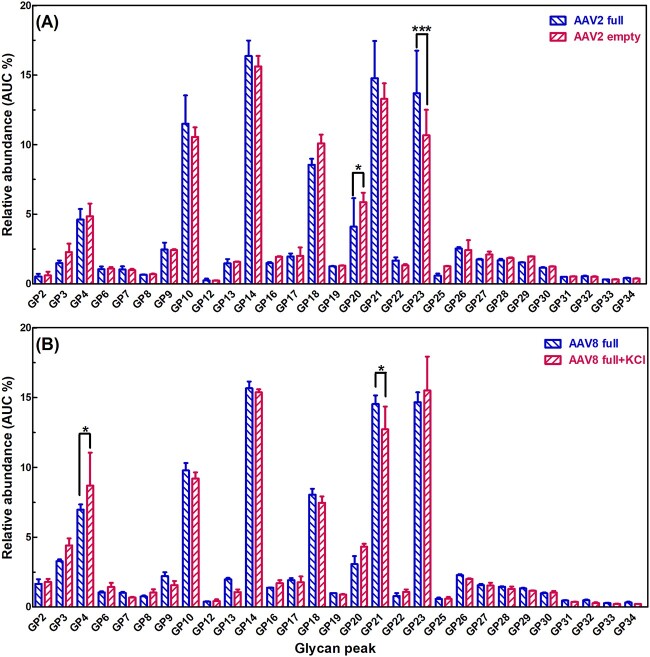
Direct comparison of the average relative abundance (%) and sample standard deviation (AUC % ± SD) of major GPs generated from biological triplicates of AAV2 serotype empty and full capsid containing cytomegalovirus (CMV)-driven green fluorescence protein (GFP) (A) and AAV8 full capsid containing CMV-driven GFP in the presence and absence from 40 mM KCl supplement (B). The comparison of biological triplicates between the treatment by two-way ANOVA is displayed, with * as *P* < 0.05, ** as *P* < 0.01, *** as *P* < 0.001. Ns (not significant) as *P* > 0.05 is not displayed. The complete dataset and statistical analysis by two-way ANOVA are shown in Supplementary Information ST2 and ST3.

To evaluate any potential variations in N-glycosylation profiles due to the encapsidation of genomic material, we prepared AAV2 empty and full capsids corresponding to the absence and presence of the transgene of cytomegalovirus (CMV)-driven green fluorescence protein (GFP) under identical cell culture condition as described in Materials and Methods. As shown in Fig. 5A, the N-glycosylation profiles of AAV2 full and empty capsids were comparable with no significant difference for most of the glycan peaks except GPs 20 (FA2G2S1) and 23 (M9). The relative abundance of GP20 (FA2G2S1) was 4.1 ± 2.0% in the AAV2 full capsid and increased slightly to 5.9 ± 0.7% in empty capsid (*P* < 0.05). The relative abundance of GP23 (M9) decreased from 13.7 ± 3.1% for AAV2 full capsid to 10.7 ± 1.8% for the empty capsid (*P* < 0.001). This modest level of variation in N-glycosylation profiles between AAV2 empty and full capsids led us to assume that the more extensive variation in N-glycosylation profiles among different AAV serotypes observed above was due to the intrinsic characteristic of the different capsids rather than the presence or absence of vector genomes.

In order to determine whether the addition of salt to the HEK Expi293F™ transfection and culturing system would affect the N-glycosylation profiles, we prepared AAV8 serotype in the absence and presence of added 40 mM KCl but keeping all other cell culture conditions identical as described in Materials and Methods. As shown in Fig. 5B, the N-glycosylation profiles of AAV8 in the absence and presence of added 40 mM KCl were comparable with no significant difference for most of glycan peaks except GPs 4 (FM3) and 21 (M8). The relative abundance of GP4 (FM3) for AAV8 increased slightly from 7.0 ± 0.4% to 8.7 ± 2.3% following the addition of 40 mM KCl (*P* < 0.05). While the relative abundance of GP21 (M8) for AAV8 decreased slightly from 14.5 ± 0.6% to 12.7 ± 1.6% following the addition of 40 mM KCl (*P* < 0.05). These results showed only a minimal variation in the N-glycosylation profile of AAV8 following the addition of 40 mM KCl.

### N-glycan subclass analysis among the AAV serotypes

**Fig. 6:**
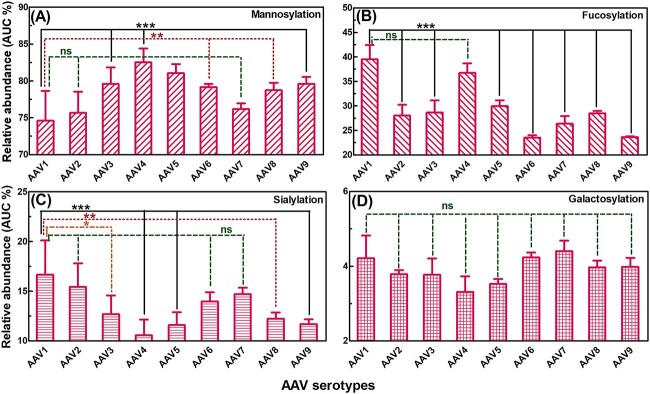
Direct comparison of average relative abundance (%) and sample standard deviation (AUC % ± SD) of the derived traits of N-glycan features among nine AAV serotypes with A), B), C), and D) representing mannosylation, fucosylation, sialylation, and galactosylation, respectively. The comparison of biological triplicates between AAV1 and the other 8 AAV serotypes by two-way ANOVA is displayed, with ns (not significant) as *P* > 0.05, * as *P* < 0.05, ** as *P* < 0.01, *** as *P* < 0.001. The formula, calculation, complete dataset, and statistical analysis by two-way ANOVA are shown in Supplementary Information ST4.

N-glycan subclasses sharing specific structural features were compared quantitatively among different AAV serotypes. This included sialylation, galactosylation, fucosylation, and mannosylation. Calculations and statistical analysis by two-way ANOVA for the complete dataset are shown in Supplementary Information (ST4—N-glycan feature calculation). Figure 6A shows the difference in mannosylation among different AAV serotypes. Mannosylated N-glycans exists at 74.6 ± 4.0% in AAV1 serotype, and there was no significant difference found between AAV1 and AAV2 or AAV7 (*P* > 0.05). There was a noticeable increase in the content of mannosylated N-glycans in AAVs 6 and 8 when compared to AAV1 (*P* < 0.01). The most significant increases in mannosylated N-glycans were observed in AAVs 3, 4, and 9 (*P* < 0.001) compared to AAV1, where the content of mannosylated N-glycans in AAV4 was as high as 82.5 ± 1.9%. Another notable feature was the relative abundance of fucosylated N-glycans that ranged from 23.6 ± 0.5% and 39.5 ± 2.9% (Fig. 6B). AAVs 1 and 4 showed the highest content of fucosylation at 39.5 ± 2.9% and 36.7 ± 2.0%. This decreased significantly for other AAV serotypes (*P* < 0.001), to as low as approximately 23.5% in the cases of AAVs 6 and 9. Additionally, sialylated N-glycans existed to a lesser content (Fig. 6C). AAV1 showed the highest content of sialylation, accounting for 16.7 ± 3.4%, which is similar to that in AAVs 2, 6, and 7 (*P* > 0.05). This decreased significantly in AAVs 4, 5, and 9 (*P* < 0.001), to as low as 10.6 ± 1.6% in AAV4. The galactosylated N-glycans were the least abundant, in the range of 3.3 ± 0.4% to 4.4 ± 0.3%, and the alterations among different AAV serotypes were negligible (*P* > 0.05, Fig. 6D).

### Classification of AAV serotypes by N-glycosylation profiles

**Fig. 7:**
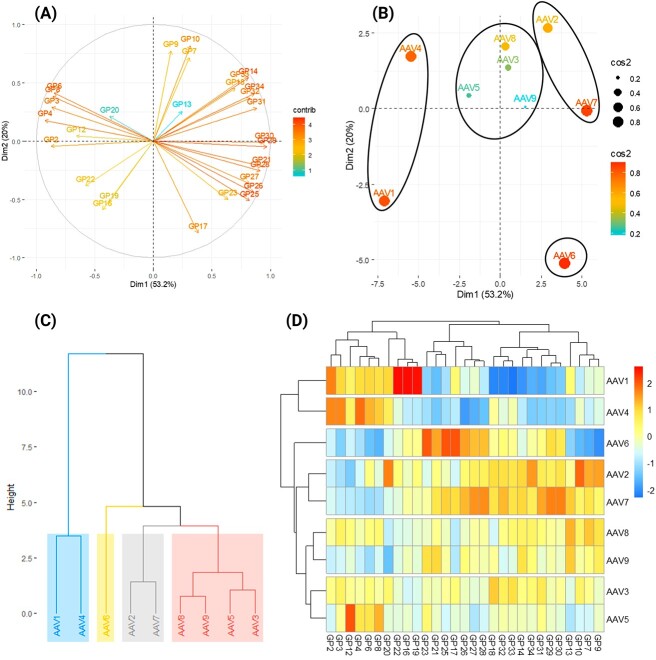
Clustering analysis for potential classification of different AAV serotypes according to the relative abundance (%) of the major GPs (excluding GPs 1, 5, 11, 15, 24, and 35 due to extremely low abundance) with A) factor analysis, B) principal component analysis, C) cluster dendrogram, and D) hierarchical clustering heatmap. A) Factor analysis plot to demonstrate correlation between the significant GP variables and dimensions. B) Principal component analysis plot to demonstrate correlation between the significant components and AAV serotypes. C) Cluster dendrogram provides visual representation of hierarchy clusters in which distances are converted into heights. D) Hierarchical clustering heatmap rows display the nine investigated AAV serotypes, and columns display the N-glycosylation profile variables. The dendrogram on the side shows the clustering of different AAV serotypes, and the dendrogram on top shows the clustering of different N-glycosylation variables.

To identify potential structural relationships between the nine AAV serotypes, unsupervised statistical cluster analysis was employed. The relative abundance (%) of 29 out of the 35 major GPs (excluding GPs 1, 5, 11, 15, 24, and 35 due to low abundance) was utilized to compare the N-glycosylation profiles between the different AAV serotypes (Fig. 7). Multivariate factor analysis was performed using the relative abundance of the 29 major GPs to classify AAV serotypes into different cohorts (Fig. 7A). The nine AAV serotypes may be grouped into four major clusters. AAVs 3, 5, 8, and 9 exhibited the closest similarity and formed one cluster. AAVs 2 and 7 formed another major cluster. AAV6 itself was designated into its own cluster. While AAVs 1 and 4 formed the last major cluster (Fig. 7B). This observation was further confirmed by cluster dendrogram and hierarchical clustering heatmap as shown in Fig. 7C and D, respectively. The most common classification method developed for AAV serotypes is based on neighbor-joining tree to form a clade or clone (Gao et al. 2004; Mietzsch et al. 2021). However, there is a substantial difference between our classification and that of the clade method. A reason for this is that the clade method classifies the AAV serotypes based on genomic and amino acid sequences that understandably are different from the changes in N-glycosylation profiles that we analyzed.

Subsequently, we tried to correlate our current classification with known host cell receptors and tissue tropism, although it is to be noted that the availability of relevant data is very limited as shown in Fig. 1D. However, no clear correlations were found between our N-glycan classification of AAV serotypes and their apparent tissue tropism. Nevertheless, we are able to distinguish AAV serotypes by clusters based on N-glycosylation profiles, that offers an alternative method to classify different AAV serotypes.

## Discussion

AAVs have become an important platform technology for therapeutic human gene delivery (Mueller and Flotte 2008). The hypervariable regions (HVRs) are the primary source of amino acid sequence variation (Govindasamy et al. 2013) that determine the structural dynamics and tissue specificity of a particular AAV serotype (Rayaprolu et al. 2013). Post translational modifications (PTMs) of AAV capsids play a significant role in defining viral properties (Mary et al. 2019). As one of the most prominent PTMs, glycosylation may impact the virus-host interactions (Miller et al. 2021). Consequently, the glycans on AAV capsids may also influence tissue specificity. However, the glycosylation of AAV capsids has been largely unexplored. Only the most abundant N-glycan species have been identified for AAV2 capsid protein (Murray et al. 2006; Mary et al. 2019). The N-glycosylation profile of AAV8 capsids have also been analyzed but these samples could have contained host cell glycoproteins because of the method of purification by density gradient centrifugation (Aloor et al. 2018).

The motivation of our current work was to provide insight into the N-glycosylation features of available AAV serotypes by providing a greater understanding of the structure of AAVs that might lead to a better understanding of their mechanism of action. By using a simplified and streamlined workflow for high-throughput and high-sensitivity N-glycan preparation and profiling (Xie et al. 2021), we profiled the N-glycosylation for a selection of nine AAV serotypes with good reproducibility. We were able to identify and quantify a total of 35 major glycan peaks (GPs) containing 81 N-glycan structures. The N-glycosylation profiles for the nine investigated AAV serotypes were comparable, although the relative abundance (%) for some GPs vary substantially (Fig. 2 and Supplementary Information ST1 and ST2). To our knowledge, this is the first time that the compositional N-glycosylation profiles of a large range of widely used AAV serotypes have been characterized and compared in detail.

Density gradient centrifugation enables the separation of macromolecules and viruses based on their mass density (Vinograd and Hearst 1962; Vinograd et al. 1963), but with limited specificity and low purity. Therefore, samples may contain contaminants, that are present in the final step (Strobel et al. 2015; Grieger et al. 2016). In our current study, affinity chromatography (Rodriguez et al. 2020) was employed to purify all the AAV serotypes using AAVX affinity resin. The affinity ligands on the resin have recombinant single-domain antibody fragments (VHHs) comprising three complementarity-determining regions (CDRs) that form the antigen-binding domain specific for AAVs. This ensures high specificity for the AAVs and therefore offers highly effective clearance of impurities, high and low molecular weight species, host cell proteins (HCPs) and DNA. It enables purification of a wide range of AAV serotypes with very high purity and yield due to its high selectivity, binding capacity, and resin reusability.

A certain percentage (approximately 30%) of empty capsids lacking desired vector genomes may co-elute with the AAV full capsids (Florea et al. 2023). By taking advantage of the overall charge difference between AAV empty (isoelectric point: 6.3) and full (isoelectric point: 5.9) capsids (Venkatakrishnan et al. 2013), anion exchange chromatography (AEX) (Aebischer et al. 2022) was employed for the separation of AAV empty and full capsids. Additionally, as shown in Fig. 5A, the representative N-glycosylation profiles between AAV2 empty and full capsids were comparable with only a modest level of variation. Therefore, it is reasonable to deduce that the major variations in N-glycosylation profiles are due to the specific AAV serotype irrespective of the genetic content. Furthermore, the methods of purification ensure that the potential interference from host cell protein impurities is negligible.

The most noticeable feature in our analysis of N-glycosylation profiles on AAVs was the surprisingly high abundance of high-mannose glycans (HMGs), including FM3, M5, M6, M7, M8 and M9 (Figs 2A, 4A, and  6A, and Supplementary Information ST1, ST2, and  ST4). These glycans dominated the HILIC-FLD chromatograms, making the total content of mannosylated glycan structures within a range of 74% to 83% for the nine investigated AAV serotypes. However, the exact composition of HMGs in each AAV serotype differs substantially. Our observations on the abundance of HMGs on AAV serotypes are supported by previous research on virus—cell interactions, where the HMGs have been shown to be important in interacting with the host cell surface attachment factors, that may contain sulfated glycosaminoglycans (GAGs) and sialic acid (Li et al. 2017; Tortorici et al. 2019; Ogharandukun et al. 2020; Robson 2020; Zhao et al. 2021). Additionally, it is well known that the envelope glycoproteins of human immunodeficiency virus (HIV) and hepatitis C. virus (HCV) have high proportion of HMGs (Hamorsky et al. 2019; Dent et al. 2021). HMGs have been observed to interact with mannose-specific receptors expressed on dendritic cells in vitro (Vander Kooi et al. 2022) as well as various other cell types (Otter et al. 1992; Wilt et al. 1999; Szolnoky et al. 2001; Linehan 2005; Izumi et al. 2017). High mannose glycoforms have also been well documented to have a significant impact on the therapeutic profile and pharmacokinetics of glycoproteins or antibodies. They enhance antibody-dependent cellular cytotoxicity (ADCC) activity, decrease complement-dependent cytotoxicity (CDC) activity, increase binding affinity to CD16 (also known as FcγRIII) receptor, decrease binding affinity to FcγRIIA and IIB receptors of therapeutic antibodies, and induce faster serum clearance rate (Goetze et al. 2011; Yu et al. 2012). Although it is likely that the blood clearance of AAV may be regulated by many factors, the high content of HMGs may contribute to the fast blood clearance although it may vary between different serotypes (Zincarelli et al. 2008; Kotchey et al. 2011; Duan 2016).

Another observation on the N-glycosylation profiles of different AAV serotypes was the relatively modest abundance of sialylated glycans, in the range of 10% to 17% (Fig. 6C and Supplementary Information ST4). One explanation for this apparent low level of sialylation may be in the nature of interaction between the AAVs and the host cell surface. Carbohydrates on host cell surface, especially N- or O-linked α (2–3) or (2–6) sialic acids, heparan sulfate proteoglycans (HSPGs), and galactose, are well recognized as the primary receptors for AAVs (Summerford and Samulski 1998; Kaludov et al. 2001; Shen et al. 2011), while laminin receptor (LamR) has been identified as a common secondary receptor on the host cell surface (Akache et al. 2006).

Sialic acids are a highly diverse family of monosaccharides that serve as terminal residues of N- and O-linked glycoproteins and glycosphingolipids (gangliosides). They create a negative charge on the surface of cell membranes at physiological pH, primarily due to their carboxyl groups (Eylar et al. 1962; Traving and Schauer 1998; Varki 2008). Additionally, HSPGs comprise covalently attached heparan sulfate (HS) glycosaminoglycan (GAG) chains which also result in a strong negative charge density (Bernfield et al. 1999; Tumova et al. 2000; Hacker et al. 2005; Dreyfuss et al. 2009; Sarrazin et al. 2011). AAVs may take advantage of the electrostatic interactions, commonly observed in protein interactions (Nakamura 1996; Sinha and Smith-Gill 2002; Zhang et al. 2011; Zhou and Pang 2018), between these negatively charged residues and the positively charged basic amino acids on capsid proteins to increase their concentration at the host cell surface and consequently enhance their binding affinity to the entry receptors (Traving and Schauer 1998; Kern et al. 2003; Perabo et al. 2006; Varki 2008; Kamhi et al. 2013; Jinno and Park 2015; Shi et al. 2021). High abundance of sialylated glycans on the AAV capsid may potentially induce significant electrostatic repulsion effects as well as steric hindrance, which may potentially repel AAVs away from the cell surface receptors, and consequently reduce the binding affinity. This has been supported by previous research where the presence of negative charges is deleterious for functional binding of AAV2 to negatively charged heparin/HSPG, while positive charges facilitate this interaction (Perabo et al. 2006). This applies to other biotherapeutic glycoprotein or antibodies as well, where higher levels of sialylation reduce ADCC and affect target binding (Lin et al. 2015; Kellner et al. 2017; Boune et al. 2020; Branstetter et al. 2021). However, the N-glycosylation profiles of AAVs were shown to consist predominantly of HMGs, with sialylated glycans at much lower content. Thus, for the AAV capsids, the potential electrostatic repulsion effects and steric hindrance may be low and consequently may facilitate strong AAV-host cell interaction.

Fucosylation of glycans are important in mediating some molecular interactions and high binding. Fucosylated glycan moieties on cell surface are recognized as critical factors for a variety of physiological and pathological processes including cell adhesion and recognition, immune cell development and function regulation, signaling process, and angiogenesis (Becker and Lowe 2003; Ma et al. 2006; Ferrara et al. 2011; Li et al. 2018). For example, a high proportion of fucosylated N-glycans on severe acute respiratory syndrome coronavirus 2 (SARS-CoV-2) are key for mediating interaction with human angiotensin converting enzyme 2 (hACE2) through high facilitated receptor binding (Zheng et al. 2022). Previous research more relevant to AAV delivery in gene therapy demonstrated that AAV complexes conjugated with aminated L-fucose molecules resulted in enhanced gene delivery efficiencies in pancreatic cancer cells (Yoo et al. 2020). In the current study, fucosylated N-glycans presented in the range of 23%–40% in the AAV serotypes analyzed (Fig. 6B and Supplementary Information ST4). However, it is difficult to predict the scale of the effects that may be enabled by these levels of fucosylation.

By quantifying the N-glycomic profiles of nine common AAV serotypes, we have identified the key N-glycosylation features of AAVs. The most noticeable feature was the high abundance of mannosylation and relatively lower levels of fucosylation and sialylation. These N-glycosylation features may help gain a greater structural understanding of AAVs in gene delivery as a complement to amino acid sequence analysis. These analyses are fundamentally different and it is not surprising to find a substantial difference between our clustering classification of AAVs based on N-glycosylation profiles (Fig. 7) and the clade method (Gao et al. 2004; Mietzsch et al. 2021). Our analysis highlights the characteristics of N-glycosylation on AAV capsids surface, and it may play a role in the biological activities of the various AAV serotypes. We were unable to find a correlation between the N-glycan profiles and the known tissue tropism of the individual AAV serotypes. However, future research focusing on glycoengineering to remove, trim, and modulate glycans may provide evidence of the correlation between AAV capsid N-glycosylation and its biological functions (Ma et al. 2020; Schjoldager et al. 2020; Dammen et al. 2022; Zhong et al. 2022).

Naturally, there are some limitations to our study. (1) We applied N-glycosylation analysis to the 9 AAV serotypes that were available to us at the start of our study. There are now at least 13 distinct primate AAV serotypes and numerous variants that have been developed for potential clinical use, and there may well be more new serotypes developed in the future. Clearly, it is not possible to investigate all these AAV serotypes. (2) There is no defined VP 1/2/3 stoichiometry for AAVs, suggesting the assembly may be stochastic (Snijder et al. 2014). The exact ratio of VP 1/2/3 is commonly estimated to be 1:1:10 (Xie et al. 2002; Walters et al. 2004; Nam et al. 2007; Strauss and Strauss 2008). VP2/VP3 and VP3-only capsids have been found to be non-infectious, whereas VP1 maintains infectivity (Grieger et al. 2007). Therefore, analysis of the glycosylation features of individual VP1, VP2, and VP3 may reduce heterogeneity and better correlate glycomic profiles with potential biological activities. (3) Furthermore, glycoengineering to remove, trim, and modulate N-glycans to prepare AAVs with specific glycoforms would be extremely valuable to demonstrate the relationship between glycosylation feature and biological function. Such an understanding would enable the design of specific AAV structures to meet targeted clinical activity and aid future rational design AAV serotypes.

Despite these limitations, identification and quantification of the overall N-glycomic profiles provide detailed comparative N-glycan compositional and structural information for the variant AAV serotypes. To our knowledge, this is the first time that N-glycomic profiles of up to nine AAV serotypes have been investigated in such detail. Despite considerable research on the biological properties of AAVs that includes pathogenicity, immunogenicity, and tissue tropism, there have been limited structural investigations particularly over the N-glycomic profiles of AAV capsids. The N-glycosylation profiles of AAVs may be a critical quality attribute (CQA) and monitored during any scale of bio-manufacture for clinical use. It is believed that the current study of quantitative N-glycomic profiling of different AAV serotypes will lay a solid foundation for better understanding of AAVs as a gene delivery system.

## Supplementary Material

20230912_AAV_capsid_glycosylation_supplementary_information_cwad074

## Data Availability

The data underlying this article are available in the article and in its online Supplementary Information.
